# *In Vivo* Imaging of Schistosomes to Assess Disease Burden Using Positron Emission Tomography (PET)

**DOI:** 10.1371/journal.pntd.0000827

**Published:** 2010-09-21

**Authors:** Nicolas Salem, Jason D. Balkman, Jing Wang, David L. Wilson, Zhenghong Lee, Christopher L. King, James P. Basilion

**Affiliations:** 1 Department of Radiology, Case Western Reserve University, Cleveland, Ohio, United States of America; 2 Department of Radiology, University Hospitals Case Medical Center, Cleveland, Ohio, United States of America; 3 Department of Biomedical Engineering, Case Western Reserve University, Cleveland, Ohio, United States of America; 4 Center for Global Health and Disease, Case Western Reserve University, Cleveland, Ohio, United States of America; 5 Veterans Affairs Medical Center, Cleveland, Ohio, United States of America; 6 NFCR Center for Molecular Imaging, Case Western Reserve University, Cleveland, Ohio, United States of America; Uniformed Services University, United States of America

## Abstract

**Background:**

Schistosomes are chronic intravascular helminth parasites of humans causing a heavy burden of disease worldwide. Diagnosis of schistosomiasis currently requires the detection of schistosome eggs in the feces and urine of infected individuals. This method unreliably measures disease burden due to poor sensitivity and wide variances in egg shedding. *In vivo* imaging of schistosome parasites could potentially better assess disease burden, improve management of schistosomiasis, facilitate vaccine development, and enhance study of the parasite's biology. *Schistosoma mansoni* (*S. mansoni*) have a high metabolic demand for glucose. In this work we investigated whether the parasite burden in mice could be assessed by positron emission tomography (PET) imaging with 2-deoxy-2[^18^F]fluoro-D-glucose (FDG).

**Methodology/Principal Findings:**

Live adult *S. mansoni* worms FDG uptake *in vitro* increased with the number of worms. Athymic nude mice infected with *S. mansoni* 5–6 weeks earlier were used in the imaging studies. Fluorescence molecular tomography (FMT) imaging with Prosense 680 was first performed. Accumulation of the imaging probe in the lower abdomen correlated with the number of worms in mice with low infection burden. The total FDG uptake in the common portal vein and/or regions of elevated FDG uptake in the liver linearly correlated to the number of worms recovered from infected animals (R^2^ = 0.58, *P*<0.001, n = 40). FDG uptake showed a stronger correlation with the worm burden in mice with more than 50 worms (R^2^ = 0.85, *P*<0.001, n = 17). Cryomicrotome imaging confirmed that most of the worms in a mouse with a high infection burden were in the portal vein, but not in a mouse with a low infection burden. FDG uptake in recovered worms measured by well counting closely correlated with worm number (R^2^ = 0.85, *P*<0.001, n = 21). Infected mice showed a 32% average decrease in total FDG uptake after three days of praziquantel treatment (*P* = 0.12). The total FDG uptake in untreated mice increased on average by 36% over the same period (*P* = 0.052).

**Conclusion:**

FDG PET may be useful to non-invasively quantify the worm burden in schistosomiasis-infected animals. Future investigations aiming at minimizing non-specific FDG uptake and to improve the recovery of signal from worms located in the lower abdomen will include the development of more specific radiotracers.

## Author Summary

Schistosomiasis is a well studied parasitic disease that is far from eradication despite the development of an effective treatment. The lack of an efficacious vaccine and high re-infection rates after treatment are major factors in its intractable worldwide prevalence. A non-invasive imaging technique like positron emission tomography (PET) could give clinicians and researchers a quantitative and visual tool to characterize the worm burden in infected individuals, determine the efficacy of a candidate vaccine, and provide information about parasite migration patterns and basic biology. We are therefore proposing the novel application of PET imaging to schistosomiasis in order to advance the management and research of this infectious disease. Herein, we demonstrate that schistosome parasites take up 2-deoxy-2[^18^F]fluoro-D-glucose (FDG). FDG uptake in regions adjacent to or within the liver linearly correlate with the worm number in infected mice, but the correlation was stronger in mice with high infection burdens. We anticipate that this research is a first step in the development of more specific radiotracers optimized for schistosomiasis, and will eventually translate to human studies.

## Introduction

Schistosomiasis is a disease of chronic morbidity affecting the liver, mesentery, and urogenital tract of infected individuals, caused by the parasitic trematode worms, schistosomes. In certain endemic regions like China, the increasing focus on antischistosomal chemotherapy over the past 30 years has dramatically decreased infection rates [1], [2]. However, global prevalence still exceeds 207 million people because of post-treatment re-infection and inadequate control measures, leaving over 779 million people at risk for future infection [3]. In addition, researchers familiar with the true morbidity of schistosomiasis have indicated a greater disability-adjusted life year (DALY) than was previously thought, estimating up to a 70 million DALY burden [4]. Potential resistance to praziquantel, the current treatment standard also became an emerging concern in 1991 when an initial trial in Senegal reported low cure rates, although these results have not been confirmed [2]. A reduced susceptibility to praziquantel among certain naturally occurring Kenyan isolates of *Schistosoma mansoni* (*S. mansoni*) was also reported [5].

Eradicating schistosomiasis will require a multifaceted approach that emphasizes the development of new drugs, vaccines [6] and better diagnostic methods [7], [8]. Currently, such advances are stymied by the inability to accurately assess worm burden in infected humans. Stool examination and urine filtration are still the techniques of choice for diagnosis and testing candidate drug efficacy. These methods are unfortunately time-consuming, labor-intensive, costly, and unreliable because of daily variability and poor sensitivity [7], [9]–[12]. For example, when host egg excretion falls below 100 eggs per gram of stool (equivalent to an infection burden below 40 worms), it is increasingly difficult to accurately assess the disease burden [7], [8], [13]. These shortcomings may overestimate treatment efficacy and delay the detection of praziquantel-resistant strains. Immunodiagnostic techniques are becoming increasingly popular because of their high sensitivity and ease of use, but they rely on circulating antibodies, which have a significant time-delay with respect to infection and treatment [7], [14]–[16]. Furthermore, immunodiagnostic measures are of little use in vaccination trials because subjects have elevated antibody levels. In order to expedite research, vaccine development, and diagnosis of schistosomiasis, these challenges necessitate the development of better tools for determining infection burden.

In the past decade, small animal molecular imaging probes have achieved considerable success for non-invasively assessing disease status. Fluorescence molecular tomography (FMT) using near-infrared imaging probes activated by the abundant cathepsins in the schistosomes' digestive tract can quantify the worm burden in murine models of schistosomiasis [17]–[19]. However, this technology currently cannot be applied to human subjects. Positron emission tomography (PET) imaging is useful in measuring cellular metabolism and has emerged as a key non-invasive imaging tool for the diagnosis, staging and evaluation of treatment response for cancer in human patients [20], [21]. We hypothesized that this *in vivo* molecular imaging technology may also provide a tool to detect and quantify adult helminth parasites.

Bueding performed the first worm physiology experiments in 1950 and found schistosomes to be demanding consumers of glucose, metabolizing their dry body weight in roughly four hours [22]. We reasoned that 2-deoxy-2[^18^F]fluoro-D-glucose (FDG), a glucose analogue clinically used as a PET radiotracer for glucose metabolism, could be useful for imaging adult schistosomes. The following work assesses the usefulness of FDG PET to quantify *S. mansoni* worm burden and treatment efficacy in a mouse model of schistosomiasis.

## Materials and Methods

### *In vitro* FDG Uptake

Unless mentioned otherwise, chemicals and reagents were obtained from Invitrogen (Carlsbad, Ca). *In vitro* uptake experiments in adult schistosomes were conducted using both female and male, paired and unpaired worms perfused from 5-weeks post-infection CD-1 outbred mice (see below for infection protocol). Perfused worms were washed 4 times in 1.5 ml eppendorf tubes with 1x phosphate-buffered saline (PBS) in which glucose was added at a concentration of 1 g/L. Tubes were arranged in triplicate, each with either 1 or 5 worms per tube. Six hundred microliters of Dulbecco's Modified Eagle Medium (DMEM) low glucose medium (1 g/L) containing 150 µCi of FDG was added to each tube and incubations were carried out at room temperature. Clinical grade FDG was supplied by PETNET solutions/Siemens (Knoxville, TN) with a specific activity larger than 2,000 Ci/mmol. After one hour, each tube was washed four times using cold 1x PBS with 1 g/L of glucose. The tubes were then placed in a Wallac 1282 CompuGamma well counter (Perkin Elmer Life Science; Waltham, Ma) to measure gamma rays emitted from ^18^F decay in counts per minute (cpm). In some experiments, 20 µl of 10% sodium iodide and 5% iodine was added to 250 µl of PBS containing single adult worms for 5 minutes. The killed worms were washed twice in PBS prior to adding the FDG.

### Animals

Six-week old female athymic nude mice (nu/nu) were anesthetized using a 500-µl solution of avertin (1∶1 tert-amyl alcohol:tribromoethanol; Sigma Aldrich; St-Louis, MO) diluted to 2.5%. Mice were infected percutaneously with the aim to obtain a wide range of parasite burdens. The animals were kept in isolation facilities for 5–6 weeks until imaging. To minimize background fluorescence during FMT imaging studies, mice were switched to a low-fluorescence diet (Harlan laboratories; Indianapolis, IN) three days prior the experiments. Mice were deprived of food overnight before imaging experiments. All animal studies were conducted in accordance with the regulations and guidelines set forth by the Institutional Animal Care and Use Committee (IACUC) at Case Western Reserve University.

### Fluorescence Molecular Tomography and Magnetic Resonance Imaging Studies

Eight infected and two uninfected (control) mice were injected in the tail vein with 2 nmol of Prosense 680 (Visen Medical Inc.; Bedford, MA) diluted in 100 µl of sterile 1x PBS. Twenty-four hours after injections, mice were anesthetized with isoflurane (Aerrane; Baxter; Deerfield, IL), placed in imaging cassettes and imaged on a FMT 2500 imaging system (Visen Medical Inc.; Bedford, MA) using the 685 nm channel. Anesthesia was maintained with 1.5% isoflurane and oxygen (1 L/min) flowing within the imaging chamber throughout the acquisition. After FMT imaging, wells on the half of the imaging cassette were filled with a solution of 0.05% magnevist (gadopentetate dimeglumine; Bayer Healthcare Pharmaceuticals; Montville, NJ) diluted in distilled water for co-registration purposes. The cassette was transferred to a magnetic resonance imaging (MRI)-compatible bed and imaged on a Biospin 7.0T MRI scanner (Bruker; Billerica, MA). T2-weighted images using a multi-slice multi-echo (MSME) sequence were acquired, producing a set of coronal images (repetition time (TR) = 1250 ms, echo time (TE) = 15 ms, flip angle = 90°, voxel size = 0.0312×0.0312×0.05 cm). Anesthesia was maintained as described above. The animals were allowed to recover from anesthesia in their cages for at least one hour before microPET (μPET) imaging. FMT and MRI images were co-registered using COMKAT, a Matlab-based general-purpose compartment modeling software tool [23].

### μPET imaging

Thirty-five infected and four uninfected (control) mice were used in the μPET imaging studies (four of the infected mice were scanned twice at an interval of three days and ten other mice had been scanned with FMT and MRI). One hundred minutes before μPET imaging, mice were anesthetized with isoflurane mixed with oxygen via isolated chambers, titrating to a respiratory rate of one breath per second. Periorbital injections of FDG dissolved in 100 µl of sterile saline were administered 90 min before imaging at a target dose of 25 µCi per gram of body weight. Mice were kept under anesthesia with isoflurane until μPET imaging to minimize muscular uptake of the tracer. Twenty minutes before imaging, a 26 gauge fluorinated-ethylene-propylene (FEP) monoject veterinary I.V. catheter coated with an inert lubricant was inserted into the bladder through the urethra. Urine was removed 5 min prior to imaging with the help of gravity to minimize imaging artifacts caused by high FDG concentration in the bladder. μPET imaging was carried out using a Siemens Concord R4 microPET system (Siemens Solutions, Knoxville, TN). The imaging protocol consisted of a 45-min emission scan followed by a 10-min transmission scan using the ^57^Co point source for attenuation correction. The images were reconstructed using a 2D ordered subset expectation maximization (OSEM) algorithm, yielding a volume of 128×128×63 voxels with a voxel size of 0.85×0.85×1.21 mm and a spatial resolution of approximately 1.84 mm [24]. The μPET scanner was calibrated prior to the imaging studies using a phantom with a known radioactivity concentration measured by a dose calibrator. Verification scans confirmed that the relative error in quantifying the true radioactivity (as measured with a dose calibrator) in a phantom by measuring it using μPET imaging was less than 5%.

### Praziquantel Treatment

Six infected mice were treated with praziquantel using oral gavage. Praziquantel was diluted in 1x PBS, and titrated to 250 mg/kg body mass. Treatments were administered 72, 48, and 24 hours prior to re-imaging. μPET imaging was carried out as described above before and after treatment.

### Worm Perfusion

Perfusions were conducted using a 5-ml syringe of citrate solution infused through the right heart ventricle after mice were euthanized with a sodium pentobarbital cocktail. The portal vein was ruptured and the perfusate was collected in a sterile culture plate. Worms and worm pairs were counted and transferred to a 1.5 ml eppendorf tube. Background radioactive signal was washed from worms using an isotonic glucose solution before measuring the radioactivity levels by well counting.

### Cryomicrotome Imaging

To localize schistosomes in our mouse model of *S. mansoni* infection, two infected mice (one of which had been imaged with FMT, MRI and μPET) were fixed in OCT, flash frozen in liquid nitrogen and stored at −80°C. The frozen blocs were imaged using a cryomicrotome imaging system developed in house [25]. Bright field color images were collected at 15.6-µm resolution and the thickness of each section was 40 µm.

### Autoradiography

One infected mouse died approximately 60 min after FDG injection and was therefore not imaged. The liver and digestive tract were rapidly removed and exposed for 30 min to a Molecular Dynamics mounted storage phosphor screen (General Electric Healthcare; Piscataway, NJ) for autoradiography. After exposure, the phosphor screen was imaged with a Typhoon imaging system (General Electric Healthcare; Piscataway, NJ).

### Microscopy

The livers of 6 mice were removed after perfusion and kept overnight in paraformaldehyde. The livers were transferred to a 30% sucrose solution overnight. Fixed tissues were embedded in OCT and kept at −80°C. Several 10 µm-thick sections from different areas of the liver were mounted on glass slides using a CM 3050 S cryostat (Leica; Bannockburn, IL) for hematoxylin and eosin (H&E) staining. Perfused worms were also directly mounted on glass slides after FMT and PET imaging. A DM 4000B microscope (Leica; Bannockburn, IL) equipped with a Cy5 filter (663–738 nm band-pass) was used to image all sections. Exposure times for the acquisition of near-infrared images of Prosense 680-labeled worms were set to 50 ms and bright field images were acquired to register fluorescent images with soft tissues using the QCapturePro software (QImaging Corporation; Surrey, BC, Canada).

### Quantitative and Statistical Analyses

Three-dimensional regions of interest (ROIs) were drawn around the lower abdomen of the mice imaged with FMT to exclude background signal from the liver. Voxels with values greater than two times the average signal in the lower abdomen of the control mice were used for quantification as previously described [17]. Adjusting the display intensity when needed, ROIs were manually drawn around the portal vein and, if present, around areas of increased FDG uptake within the liver parenchyma using ASIPro VM 6.3.3.0 (Siemens Solutions, Knoxville, TN). In some cases, the internal organs had shifted as a result of animal manipulations prior to imaging and the location of the ROIs was determined using the best judgment from the user. Areas of high FDG uptake within the small intestines or colon as determined by careful examination of multiple adjacent sections were excluded. All radioactivity measurements were decay-corrected to the scan start time for comparison. Statistical data were generated using Origin 8.1 (OriginLab; Northampton, MA). Unpaired and paired *t*-tests were used to examine the differences between groups in the *in vitro* uptake and praziquantel treatment experiments, respectively. *P*-values≤0.05 were considered significant. The square of the Pearson's product-moment correlation (R^2^) was used to determine correlation between measured signals and worm burden.

## Results

**Figure 1:**
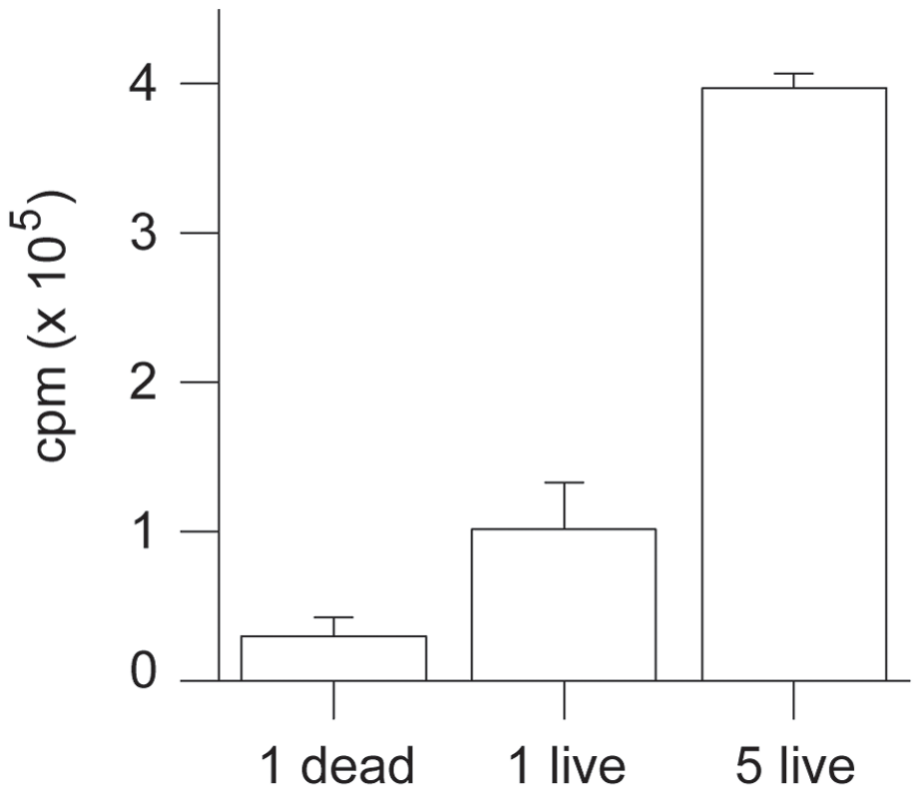
*In vitro* FDG uptake by S. mansoni. FDG uptake in counts per minute (cpm) after 60 min incubations of 5-weeks post-infection hepatic young adult-stage schistosomes (live). Iodine killed parasites (dead) of the same developmental age were used as negative controls. Error bars represent the standard deviation (n = 3 experiments each).

*In vitro* uptake of FDG was examined for adult stage *S. mansoni* (Figure 1). The uptake in single iodine-killed worms was significantly lower than that in untreated single adult worms (*P* = 0.04). The total uptake by 5 worms was 3.9 times greater than that by single worms (*P* = 0.002). Based on the 60-min incubation experiments with 5 worms, the average radioactivity per adult worm was 54 nCi, which would be sufficient for detection by μPET imaging.

**Figure 2:**
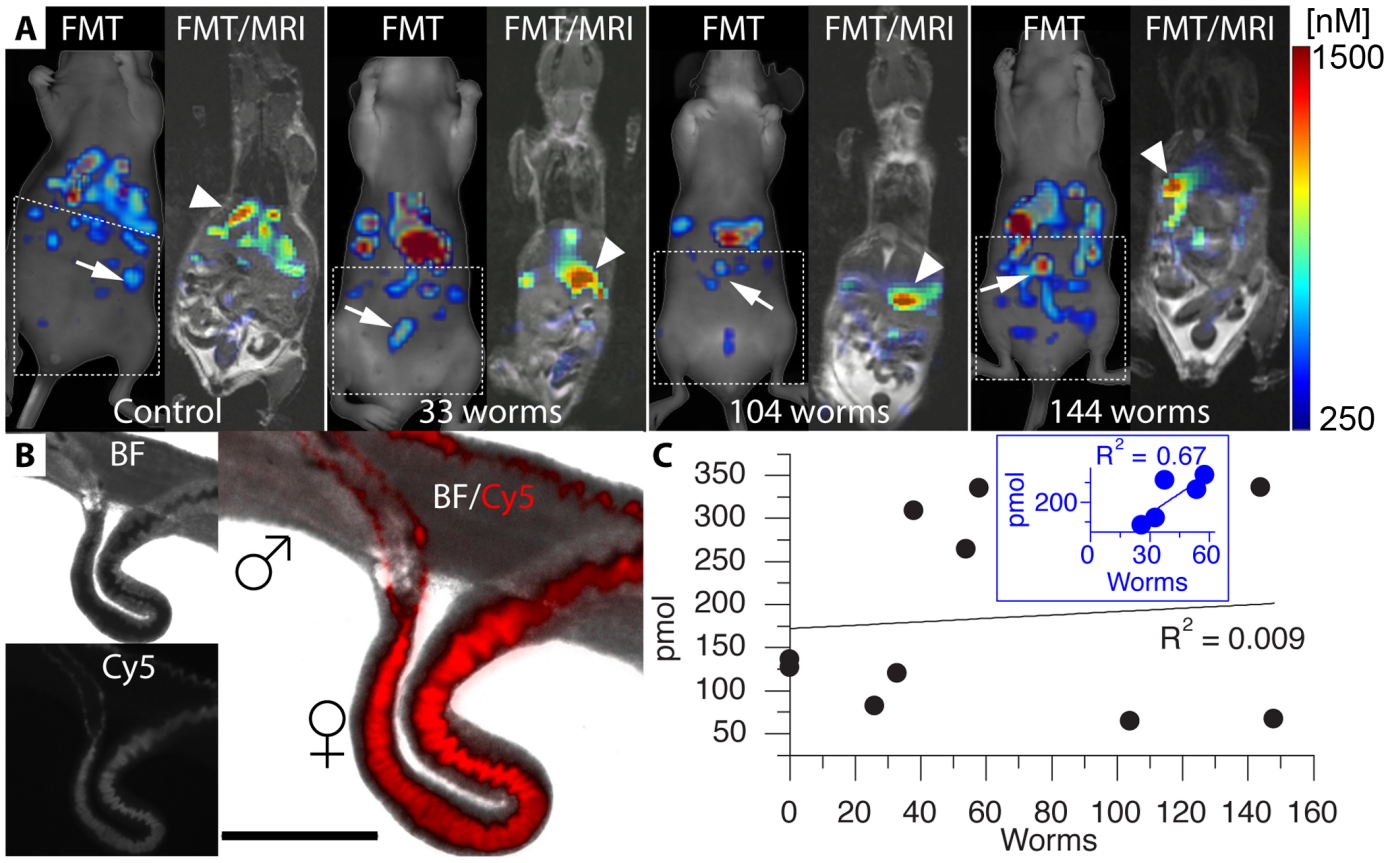
FMT and MRI of mice infected with S. mansoni. (A) Uninfected and mice with different worm burdens were scanned 24 hours after Prosense 680 injections. MRI was also performed on the same mice to provide anatomical references. Arrows indicate fluorescence in the lower abdomen. Arrowheads designate areas of fluorescence in the liver. The dotted boxes indicate the three dimensional ROIs used to quantify the probe amount. Intensity scale [250 (blue)–1500 (red) nM]. (B) Worms were collected on glass slides after perfusion for microscopy. Bright field (BF) and fluorescent images using the Cy5 filter (Cy5) and 50 ms exposures were acquired and co-registered to show that fluorescence was originating in the worms' digestive tract. A male-female pair is shown here. Scale bar = 100 µm. (C) Relationship between the total probe amount in the lower abdomen and the number of worms when considering all mice (n = 10; graph) and only infected mice with less than 60 worms (n = 5; blue inset).

Whole animal FMT and MRI scans were performed in two control and eight infected mice (5–6 weeks after infection) and the images were co-registered to provide an anatomical context for the FMT signal. Twenty-four hours after Prosense 680 injection, moderate and high fluorescence levels were observed in the mid-section of the abdomen in control and infected mice (Figure 2A). Anatomical information from MRI confirmed that the most intense fluorescence signals were originating from regions within or adjacent to the liver in infected mice. Liver expresses relatively high levels of the proteases targeted by Prosense probes. Signals of low, but variable intensity were also observed in the lower abdomen of all mice. Worms were collected after imaging and placed on a glass slide. Microscopy demonstrated high fluorescence in the digestive tracts of perfused schistosomes (Figure 2B). Adult *S. mansoni* parasites are abundant in cathepsins, which supports the basis for imaging schistosomiasis *in vivo* with a fluorescent probe activated by proteases. Total probe accumulation in the lower abdomen regions was previously found to correlate with worm burden [17]. We did not find a linear correlation between probe levels in the lower abdomen and worm burden (R^2^ = 0.009, *P* = 0.80, n = 10; Figure 2C). However, when only considering mice with less than 60 worms (a range comparable to that previously reported [17]) and excluding control mice from the analysis as was done in [17], a linear correlation between probe levels and the lower abdomen and the number of worms was observed (R^2^ = 0.67, *P* = 0.09, n = 5; Figure 2C, inset).

**Figure 3:**
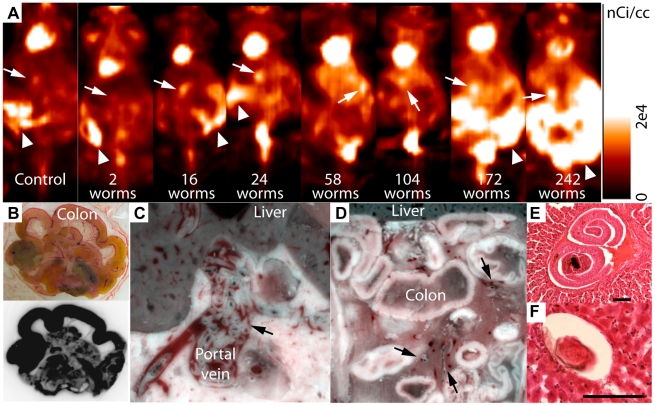
FDG-μPET imaging of mice infected with S. mansoni. (A) μPET scans were acquired in control and infected mice 90 min after FDG injection. Arrows indicate areas of moderate uptake adjacent to the liver or areas of increased uptake in the liver parenchyma around which ROIs were drawn to quantify FDG uptake (Figure S1). Arrowheads indicate areas of high uptake in the lower abdomen. The mouse with 104 worms had also been imaged with FMT/MRI (Figure 2). Note that mice were in prone position for μPET scanning and in supine position for FMT/MRI scanning. Intensity scale [0 (black)–2×10^4^ (white) nCi/cc]. (B) Photograph of the digestive tract of an infected animal that was removed 60 min after FDG injection (upper panel) and exposed to a phosphor screen for autoradiography showing high FDG uptake in the colon and diffuse FDG uptake in other parts of the digestive tract (lower panel). (C) Cryomicrotome imaging of a mouse with a heavy infection burden. A high number of worms were found in the common portal vein (arrow). (D) Cryomicrotome imaging of the lower abdomen in the same animal showed the presence of a small number of worms scattered in the mesentery (arrows). (E) Worms were found in the liver of one infected mouse that died before FDG injection. H&E staining; scale bar = 100 µm. (F) Lack of an overt inflammatory response to an ova in the liver (H&E staining). Scale bar = 100 µm.

We next performed experiments to determine if FDG-μPET imaging would allow noninvasive quantification of adult *S. mansoni* in infected mice. Four control and 35 infected mice were included in these studies. These included a subset of ten animals (2 control and 8 infected mice) that also underwent FMT imaging as described above. One of the infected mice died approximately 60 min after FDG injection and could not be imaged. In another case, the animal (high infection burden) died before FDG injection so the liver was removed and processed for histology. Four mice were scanned twice at 3-day intervals and one mouse was not perfused, but fixed in OCT after μPET imaging for whole body cryomicrotome imaging, a technique that allows microscopic examination of the entire or regional areas of the interior of the mouse [25]. Forty datasets were thus included in the quantitative studies. Nineteen mice had an infection burden below 40 worms while 17 mice carried between 50 and 242 worms. Intense radioactive signals were observed in the heart, kidneys, bladder and regions of the lower abdomen in control and infected mice (Figure 3A, arrowheads). Autoradiography of the liver and digestive tract in the mouse that died 60 min after FDG injection revealed that the areas of high radioactivity accumulation in the lower abdomen corresponded mainly to the colon and, to a lesser extent, the small intestines (Figure 3B), while the radioactivity levels in the liver were relatively low (not shown). Moderate radioactivity accumulation was observed in small regions inferior to the liver, slightly anterior to the kidneys and superior to the colon (Figure 3A, arrows). In a few mice, abnormal radioactivity accumulation was found in some regions of the liver (Figure 3A, arrows).

Cryomicrotome imaging was carried out after FDG-μPET scans in one mouse with a low (which did not undergo FDG-μPET scanning) and one mouse with a high infection burden. The worms could be readily visualized by their white soft tissue appearance and dark digestive tract containing heme pigment. In the mouse with a low infection burden, only a few worms were seen in the liver parenchyma. We did not find any schistosomes in the mesentery (not shown), but this was only in one animal. In the mouse with a high infection burden, a high number of worms were found in the common portal vein (Figure 3C, arrows). Only a few worms were seen in the liver and scattered in the mesentery (Figure 3D, arrows). H&E sections of the liver of the mouse that died before FDG injection (which had a high target infection burden) confirmed the presence of worms in branches of the portal vein in the liver (Figure 3E). The livers from five other infected mice were also processed for histology after perfusion. Examination of the H&E sections revealed the absence of worms in the liver parenchyma. Ova trapped in the liver parenchyma did not induce an overt inflammatory response (Figure 3F) indicating that regions of abnormal FDG uptake in the liver are unlikely due to granulomatous response to ova. The absence of worms from the mesentery after perfusion was confirmed by careful visual inspection.

**Figure 4:**
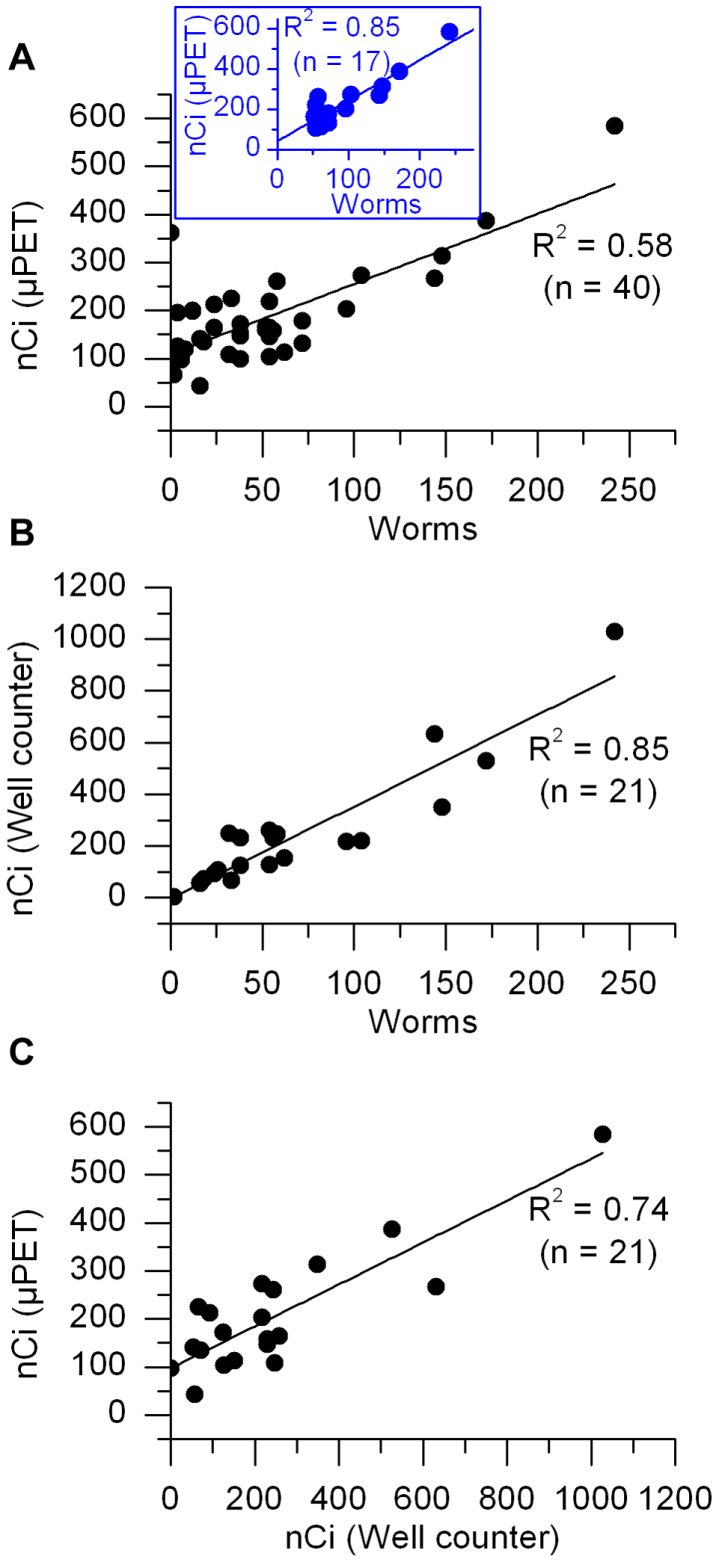
Quantitative analysis of the FDG-μPET images and well-counting data in control and infected mice. (A) Relationship between the total radioactivity within three-dimensional ROIs drawn around the portal vein and/or areas of abnormal uptake in the liver and the number of worms when considering all datasets (graph) or only datasets obtained from mice with more than 50 worms (blue inset). (B) Radioactivity levels measured by well counting as a function of the number of worms. (C) Total radioactivity measured by μPET graphed as a function of the total radioactivity measured by well counting.

Because of high background uptake in the colon and small intestines, three-dimensional ROIs were only drawn around the common portal vein, inferior to the liver and in areas of increased uptake in the liver, if present (Figure S1). A linear correlation between the total radioactivity within ROIs and the number of worms was observed (R^2^ = 0.58, *P*<0.001, n = 40; Figure 4A). The background FDG uptake in the common portal vein (y-intercept) was approximately 110 nCi. An even stronger linear relationship between total radioactivity within ROIs and the number of worms was observed in mice with more than 50 worms (R^2^ = 0.85, *P*<0.001, n = 17; Figure 4A, inset). The radioactivity levels in the schistosomes from 21 of the infected mice were directly measured by well counting after perfusion. There was a strong linear relationship between the measured radioactivity levels in perfused worms and the number of worms (R^2^ = 0.85, *P*<0.001, n = 21; Figure 4B). The total radioactivity within ROIs correlated with the measured radioactivity levels in perfused worms (R^2^ = 0.74, *P*<0.001, n = 21; Figure 4C). However, the radioactivity measured by μPET was in general less than that in the perfused worms.

**Figure 5:**
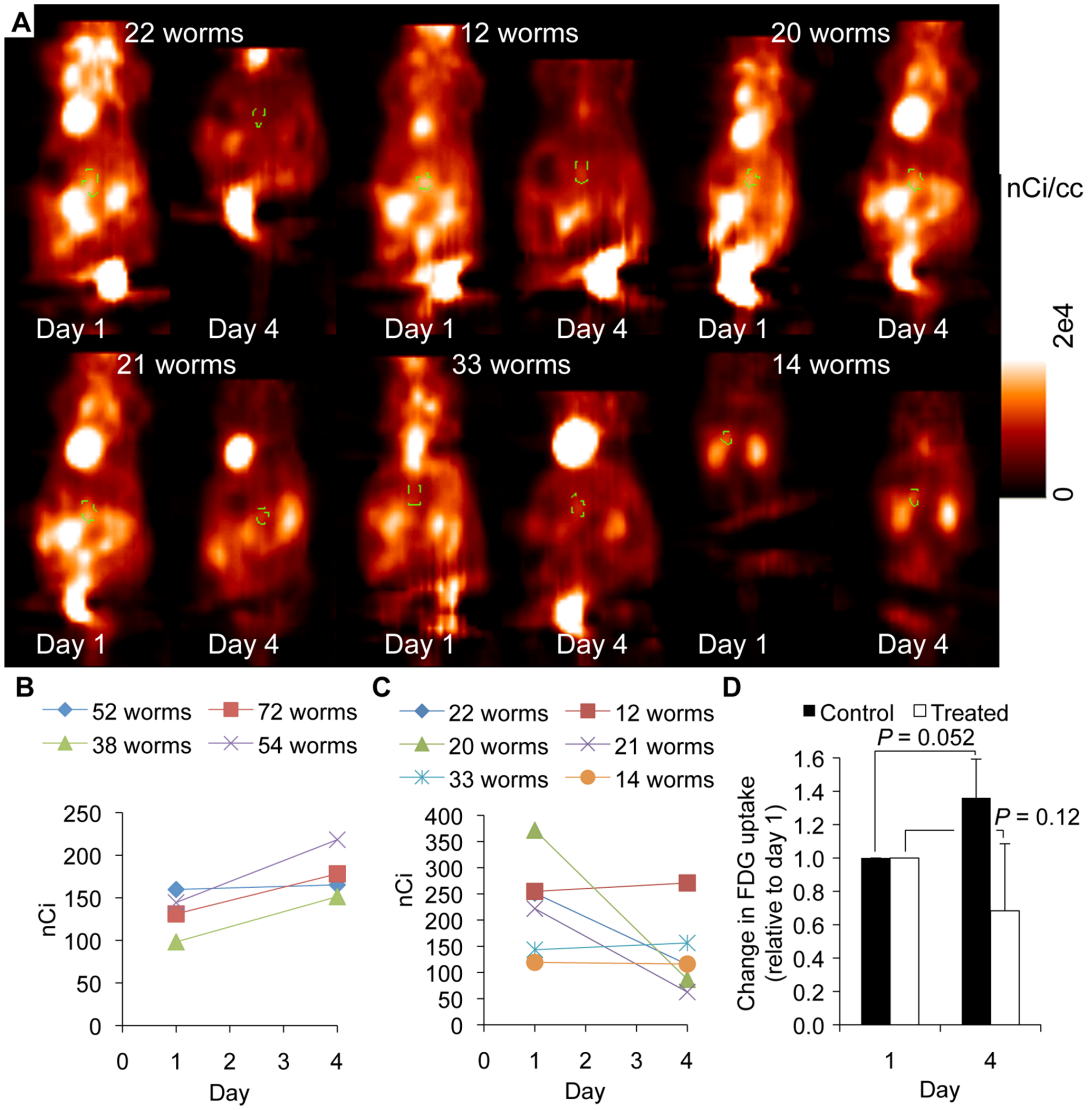
FDG-μPET imaging of praziquantel-treated mice. (A) μPET imaging performed before (Day 1) and after (Day 4) praziquantel treatment. ROIs on the coronal sections chosen for display are outlined by a green dotted line. Intensity scale [0 (black) –2×10^4^ (white) nCi/cc]. (B) Quantification of FDG uptake in the area of the common portal vein in untreated infected animals at day 1 and day 4 (* and **; Figure S1). (C) Quantification of FDG uptake in the area of the common portal vein in infected animals before and after treatment. (D) Average change in FDG uptake in treated and untreated mice relative to FDG uptake at day 1. Error bars represent the standard deviation.

Praziquantel treatment studies were finally performed to investigate the usefulness of FDG-PET for assessing treatment efficacy (Figure 5A). Four infected mice did not receive treatments and were scanned twice, at three-day intervals, serving as positive controls (* and **; Figure S1). Over the three-day period, FDG uptake in the area of the common portal vein increased by 36% on average in untreated mice (*P* = 0.052; Figure 5B & D). Comparison of uptake in the area of the common portal vein in treated mice showed a 32% average decrease in total radioactivity over the three-day treatment period, although this did not reach significance (*P* = 0.12; Figure 5C & D). The number of worms reported in Figure 5B and C were determined as described above by perfusing mice after μPET imaging on day 4. The viability of the worms was qualitatively assessed by trypan blue exclusion. Worms recovered from the mouse that exhibited the largest decreased in FDG uptake in the common portal vein (77%) were dead (did not exclude trypan blue; mouse with 20 worms in Figure 5).

## Discussion

Our current understanding of the *S. mansoni* migration cycle in the host is based on earlier studies in which radiolabeled schistosomulae were injected into mice and tracked with autoradiography [26]. Only in recent years have noninvasive imaging technologies become available for small animal studies of schistosomiasis. FMT was recently applied to schistosomiasis to quantify adult worm burden [17]. While the results showed that the number of worms in infected mice can be quantified using near-infrared probes activated by proteases, only a limited range of worm burdens was assessed and optical imaging techniques currently cannot be applied in humans. In this work, we investigated the possibility of PET imaging of *S. mansoni* in mice as this technology is readily applicable to humans. Here we show that FDG uptake in adult parasites both *in vitro* and *in vivo* correlates with parasite numbers. We also demonstrate that adult worms can be imaged *in vivo* using μPET to assess worm burden. However, our ability to quantify worm burden by FDG-μPET was limited in mice with low worm burden.

*In vitro* FDG uptake studies carried out on adult schistosomes first showed that FDG uptake in five adult worms was greater than that in single adult worms. Well counting measurements also showed that the radioactivity levels in worms recovered from infected mice after FDG μPET imaging strongly correlated with the number of worms. These results supported the hypothesis that FDG uptake quantified by μPET imaging may correlate with the worm burden. Furthermore, the lower FDG uptake in iodine-killed single worms compared to untreated single worms suggested that FDG-μPET may be useful in assessing the outcome of treatments such as praziquantel. The residual radioactivity associated with dead worms in Figure 1 likely represents the inability to remove the unmetabolized FDG following repeated washings.

To supplement the μPET data, we quantified worm burden by assaying protease activity using FMT. We first imaged infected mice 24 hours after Prosense 680 injection (Visen Medical Inc.; Bedford, MA). The images revealed high fluorescence signal coming from the liver of all mice, including uninfected animals. This was observed in a previous study unrelated to schistosomiasis comparing small molecular probes with the larger Prosense 680 and 750 probes and may be caused by the hepatic clearance of the released fluorochromes [27]. Furthermore, high levels of the proteases that activate the Prosense probes used are expressed in the liver. Because of this high background in the liver, only probe accumulation in the lower abdomen was quantified. A linear correlation between total probe amount and worm burden was observed, but only in mice with less than 60 worms. Similar results were demonstrated in another study, although only animals with less than 35 worms were included [17]. This demonstrates the presence of adult worms in the mesenteric vasculature, which was confirmed at the time the worms were recovered. The poor correlation in animals with higher worm burdens likely arises from the presence of worms retained in the common portal vein and adjacent liver parenchyma.

In contrast to FMT, FDG imaging showed relatively low FDG uptake in the liver but considerable and variable FDG uptake in the lower abdomen of all mice, regardless of the worm burden (Figure S1). Autoradiography showed that most of the radioactivity in the lower abdomen was in the colon and was not associated with schistosomes. Focal FDG uptake in normal gut can appear as false lesions in PET scans of the abdomen in humans [28]–[30]. These foci of abnormal FDG uptake can be caused by metabolism in the gut flora or by peristalsis for example. In heavily infected animals, FDG uptake in the lower abdomen may also be associated with innate inflammatory response either from products released by worms or colonic flora from compromised integrity of the gut wall. High uptake in the gut of control (uninfected) animals and low uptake in the gut of some infected animals suggests that gut motility and metabolism and a moderate immune response to schistosomiasis infection may all be factors that contribute to FDG uptake not associated with worms in the lower abdomen.

Moderate FDG uptake was observed in regions inferior to or within the liver and total radioactivity levels within these regions appeared to increase with the worm number. A linear correlation between total radioactivity within these regions and the number of worms was demonstrated across a wide range of infection burdens (0–242 worms). This correlation was especially strong in mice with high worm burden, suggesting that in heavily infected animals, most worms remain in the common portal vein and in the liver parenchyma. This was demonstrated by cryomicrotome imaging and by dissection at the time of worm recovery. We speculate that in mice with high infection burden, adults mature more slowly and thus, proportionally fewer worm pairs migrate into the mesenteric vasculature at the time the imaging studies were carried out (5–6 weeks after infection). FMT imaging, cryomicrotome imaging and direct observations at the time of perfusion showed the presence of worms in the mesenteric vasculature in heavily infected animals. However, worms in the mesenteric vasculature are in insufficient numbers to account for the large uptake of FDG observed on the PET images. The low levels of radioactivity associated with worms in the mesenteric vasculature could not be accurately and consistently detected against the high FDG uptake in the lower abdomen of some mice.

To further validate the μPET results, animals were treated with praziquantel, an anti-schistosomal drug that targets adult worms. The FDG signal measured in treated animals decreased after three days of treatment while that in untreated animals increased over the same period. The variability in change of FDG uptake in the region of the common portal vein in response to praziquantel is likely due to the use of athymic nude mice that can result in impaired parasite killing since praziquantel treatment efficacy has been shown to be dependent upon an intact host immune response [31]. Indeed variable efficacy of drug treatment was noted in trypan blue exclusion studies performed on the worms after perfusion. The analysis of the results is further complicated by the fact that the number of worms prior to the start of praziquantel treatment is unknown and thus, treatment efficacy cannot be accurately assessed. Nevertheless, these results suggest that, with some improvements in the technique and recovery of worms in the lower abdomen, PET imaging may be useful to assess treatment outcome using an individual as its own control and ROI-based analysis to assess the number of worms after treatment.

The high non-specific FDG uptake in the colon and, to a lesser extent, small intestines, that limited our ability to detect schistosomes in the mesentery in infected mice may not be such an issue when performing FDG-PET imaging studies in human patients with schistosomiasis. Methods such as colon cleansing by isosmotic solution taken the evening prior to examination, intravenous hydration with 0.45% saline and cleansing of the urinary tract and bladder during scanning were shown to yield artifact-free PET images in humans [30]. Such methods could be applied to decrease non-specific uptake in the gut and allow better detection of worms in the mesentery. Another potential background issue for these studies is that activated immune cells in granulomas formed in response to ova deposition may also uptake FDG and affect quantification of the worm burden in humans. T-cell-deficient mice were used in these experiments to avoid an immune response from the host. Previous studies reported no statistically significant difference in size between worms developing in nude mice and controls [32]. Nude mice also exhibited suppressed granuloma formation and reduced morbidity, further justifying our choice of animal model [33]. We imaged all mice 5–6 weeks post-infection, at a point in development when some worms begin egg release and most worms have matured to full adult stage. Performing these experiments in immunocompetent mice should be achievable since studies conducted in immunocompetent rats showed that treatment with a single dose of methylprednisolone resulted in decreased FDG uptake in granulomas [34]. Such a strategy could be used to distinguish between inflammatory lesions and schistosomes on FDG-PET images.

Finally, the μPET scanner used in our experiments has a spatial resolution of approximately 1.84 mm, but other small animal scanners have reported spatial resolutions as low as 0.7 mm for 10-cm detector ring radius [35], [24]. Adult schistosomes range from 0.6–1.1 cm in the thicker males (0.07 cm in diameter) and 1.2–1.6 cm for the thinner females (0.016 cm in diameter) [36]. Human PET scanners currently have a spatial resolution of approximately 5 mm, but scanners with 2 mm isotropic spatial resolution across the field of view are available [37]. While the spatial resolution of animal or human PET scanners may not be high enough to resolve individual worms, the high sensitivity of this imaging modality may allow non-invasive quantification of the worm burden in infected mice and man through region-based quantitative analysis.

In conclusion, these studies verified the hypothesis that high glucose metabolism in *S. mansoni* allows for detection with FDG-μPET and quantification of the disease burden *in vivo*. Because of the possibility to apply PET technology to humans to support the development of new diagnostic tools and for vaccine research, we believe FDG-PET imaging of schistosomiasis should be further investigated. Studies moving forward will include efforts to minimize non-specific radiotracer uptake in the gut (e.g. using antibiotic cocktails, laxatives or combinations of both) and FDG-μPET imaging studies in immunocompetent rodent models infected with schistosomiasis. Finally efforts are already underway to develop radiotracers more specific for parasites, including radiolabeled praziquantel.

## Supporting Information

Figure S1 FDG µPET imaging of control and infected mice. Three-dimensional ROIs were manually drawn around the portal vein of all animals and, when observable, in regions of increased FDG uptake in the liver. Representative coronal sections from every animal used in the quantitative study are shown. Intensity scale [0 (black) - 2×10^4^ (white) nCi/cc]. ROI outlines are shown in dotted green lines. *Infected mice imaged on day 1. **Corresponding untreated infected mouse imaged on day 4. ^†^ The ROI was drawn around a region of increased FDG uptake inferior to the liver that was not connected to the colon as determined by careful examination of all adjacent coronal sections.
(5.00 MB TIF)
