# Supplementary figures and images for: In Vivo Imaging of Schistosomes to Assess Disease Burden Using Positron Emission Tomography (PET)
Source: PLoS Negl Trop Dis. 2010 Sep 21;4(9):e827. doi: 10.1371/journal.pntd.0000827 (PMC2943464; doi:10.1371/journal.pntd.0000827)

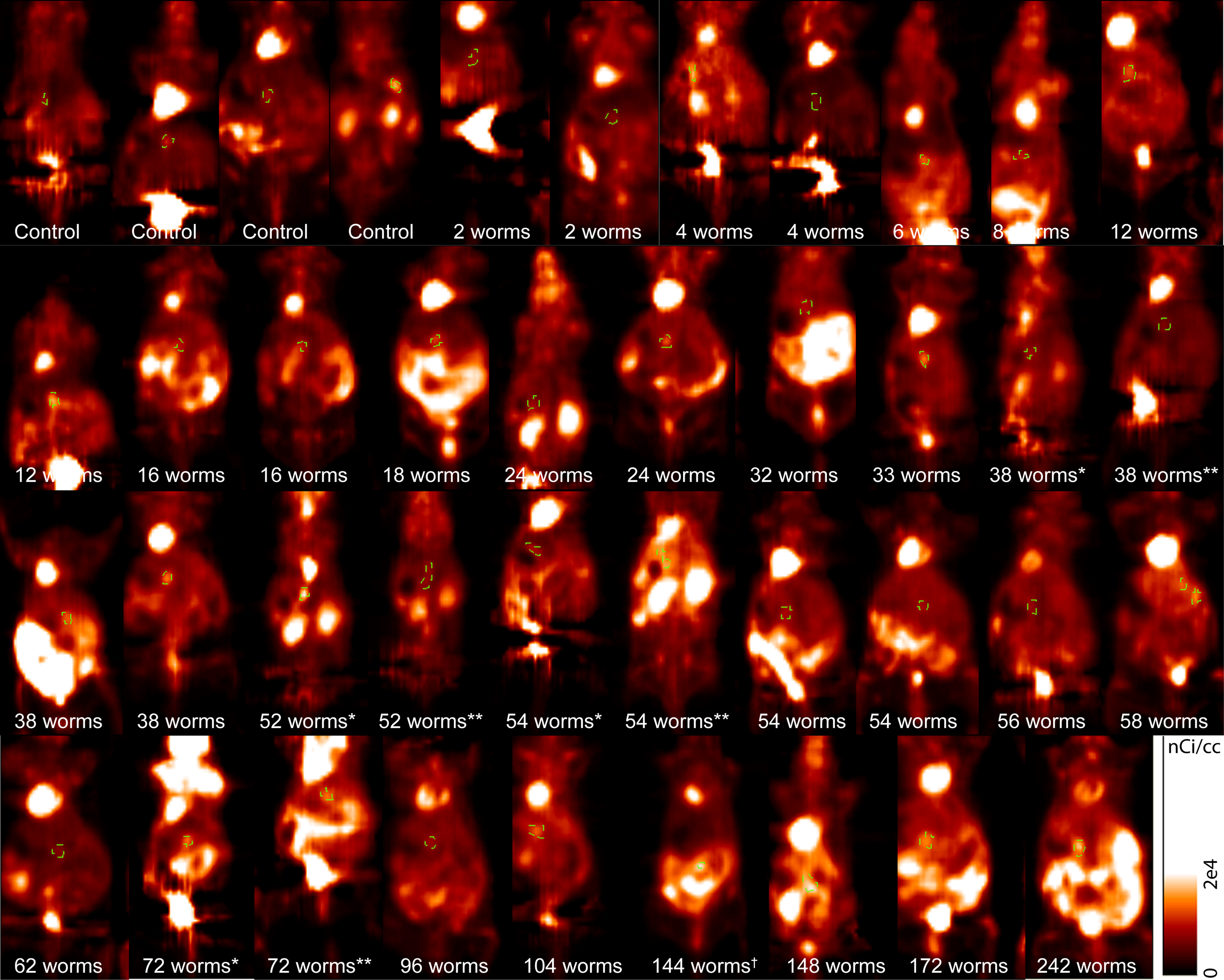

Supplement: Figure S1 — FDG µPET imaging of control and infected mice. Three-dimensional ROIs were manually drawn around the portal vein of all animals and, when observable, in regions of increased FDG uptake in the liver. Representative coronal sections from every animal used in the quantitative study are shown. Intensity scale [0 (black) - 2×10^4^ (white) nCi/cc]. ROI outlines are shown in dotted green lines. *Infected mice imaged on day 1. **Corresponding untreated infected mouse imaged on day 4. † The ROI was drawn around a region of increased FDG uptake inferior to the liver that was not connected to the colon as determined by careful examination of all adjacent coronal sections. (5.00 MB TIF) [file pntd.0000827.s001.tif]
